# Sphere-derived tumor cells exhibit impaired metastasis by a host-mediated quiescent phenotype

**DOI:** 10.18632/oncotarget.4803

**Published:** 2015-08-07

**Authors:** Anne-Marie Bleau, Carolina Zandueta, Miriam Redrado, Susana Martínez-Canarias, Leyre Larzábal, Luis M. Montuenga, Alfonso Calvo, Fernando Lecanda

**Affiliations:** ^1^ Program in Solid Tumors and Biomarkers, Division of Oncology, Center for Applied Medical Research (CIMA), University of Navarra, Pamplona, Spain; ^2^ Department of Histology and Pathology, School of Medicine, University of Navarra, Pamplona, Spain; ^3^ IdiSNA, Navarra Institute for Health Research, Pamplona, Spain

**Keywords:** stem cell, dormancy, p27, osteolysis, p38

## Abstract

The spread of lung cancer cells to distant sites represents a common event associated with poor prognosis. A fraction of tumor cells named cancer stem cells (CSCs) have the ability to overcome therapeutic stress and remain quiescent. However, whether these CSCs have also the capacity to initiate and sustain metastasis remains unclear. Here, we used tumor sphere cultures (TSC) isolated from mouse and human lung cancer models to enrich for CSCs, and assessed their metastatic potential as compared to non-CSCs. As expected, TSC overexpressed a variety of stem cell markers and displayed chemoresistance. The CSC phenotype of TSC was confirmed by their higher growth ability in soft agar and tumorigenic potential *in vivo*, despite their reduced *in vitro* cell growth kinetics. Surprisingly, the appearance of spontaneous lung metastases was strongly delayed in mice injected with TSC as compared to non-TSC cells. Similarly, this finding was confirmed in several other models of metastasis, an effect associated with a retarded colonization activity. Interestingly, such delay correlated with a quiescent phenotype whose underlined mechanisms included an increase in p27 protein and lower phospho-ERK1/2 levels. Thus, these data suggest that cells enriched for CSC properties display an impaired metastatic activity, a finding with potential clinical implications.

## INTRODUCTION

Lung cancer is the most lethal type of cancer worldwide with a 5-year overall survival rate at around 15%. Lung tumors frequently metastasize to local or distant sites such as the skeleton, an event associated with poor prognosis. But as opposed to other tumors, lung cancer is frequently diagnosed at advanced stages with the detection of already established metastases [1, 2]. In recent years, it has been suggested that a subtype of cancer cells within tumors exhibit self-renewal, multilineage differentiation and limitless potential capabilities [3]. Such cells have been termed cancer stem cells (CSCs), or tumor-initiating cells (TICs), due to their ability to generate tumors after transplantation [4]. These tumor cells also display enhanced chemoresistance [5]. However, whether CSCs also display an increased ability to initiate and sustain metastasis remains largely unknown [6, 7].

To study cancer stem cell functions, tumor sphere cultures (TSC) have been widely used as a surrogate assay [8]. These spherical structures are enriched with stem-like properties, such as the overexpression of stem cell markers, the capacity to undergo asymmetric cell division and to grow in anchorage independent conditions [9–11]. Tumor-derived cells grown from clinical samples are commonly cultured and maintained as spheres for transplantation experiments into mice [12]. They generally present a remarkable ability to initiate and sustain the development of tumors. In addition, they show enrichment in various immunophenotypic markers characteristic of tumor stem cells that usually vary among tumor types. For instance, CD44^high^/CD24^low^ markers identified breast CSCs [13], whereas the CD133^+^ fraction was enriched in glioma cells with stem cell-like properties [14]. High CD44 levels were used to isolate prostate [15], pancreatic [16], colon [17] and lung CSCs [18]. In addition, methods based on functional properties including the side population phenotype [19] and aldehyde dehydrogenase activity (ALDH) have also been extensively used [20, 21].

Previous reports have suggested an association between CSCs with the epithelial to mesenchymal transition (EMT) as well as invasive phenotypes, some key initiating steps in metastasis [22]. Recently, a direct link between ALDH activity and increased metastatic potential has been documented for breast cancer [23–25], prostate [26, 27] and osteosarcoma [28]. Similar observations were made for CD44 positive cells, showing a higher ability to induce bone [29] and lung [30] metastasis. However, whether the CSC phenotype acquired by TSC also entails metastatic traits, or rather metastatic activity could be segregated from tumor initiating ability, has not been addressed.

Studies that prone for a higher tumorigenic potential of spheres as compared to monolayer cultures are abundant. This has been demonstrated for lung cancer [31] and other solid tumors [32–38]. Yet, their role in metastatic processes, especially in their ability to colonize target organs and form secondary outgrowths, remains under debate.

In this study, we characterized the *in vivo* prometastatic ability of lung tumor cells displaying cancer stem cell properties using TSC as a method to enrich for tumor initiating cells. Using several models of metastasis, we found that despite their robust tumor-initiating activity, these cells displayed a more indolent phenotype in their colonization ability to target organs, mainly in the initial steps of micrometastasis at the target organ.

## RESULTS

### Tumor sphere cultures (TSC) overexpress stem cell markers

To determine the prometastatic activity of TSC, we used two different cell models. First, murine Lacun.3 cells were obtained from a chemically-induced lung adenocarcinoma developed in mice; it is an aggressive cell line that forms spontaneous metastases in different organs [39]. Second, we used the human lung cancer cell line H460 that develops spontaneous bone metastases in athymic nude mice [40].

**Figure 1:**
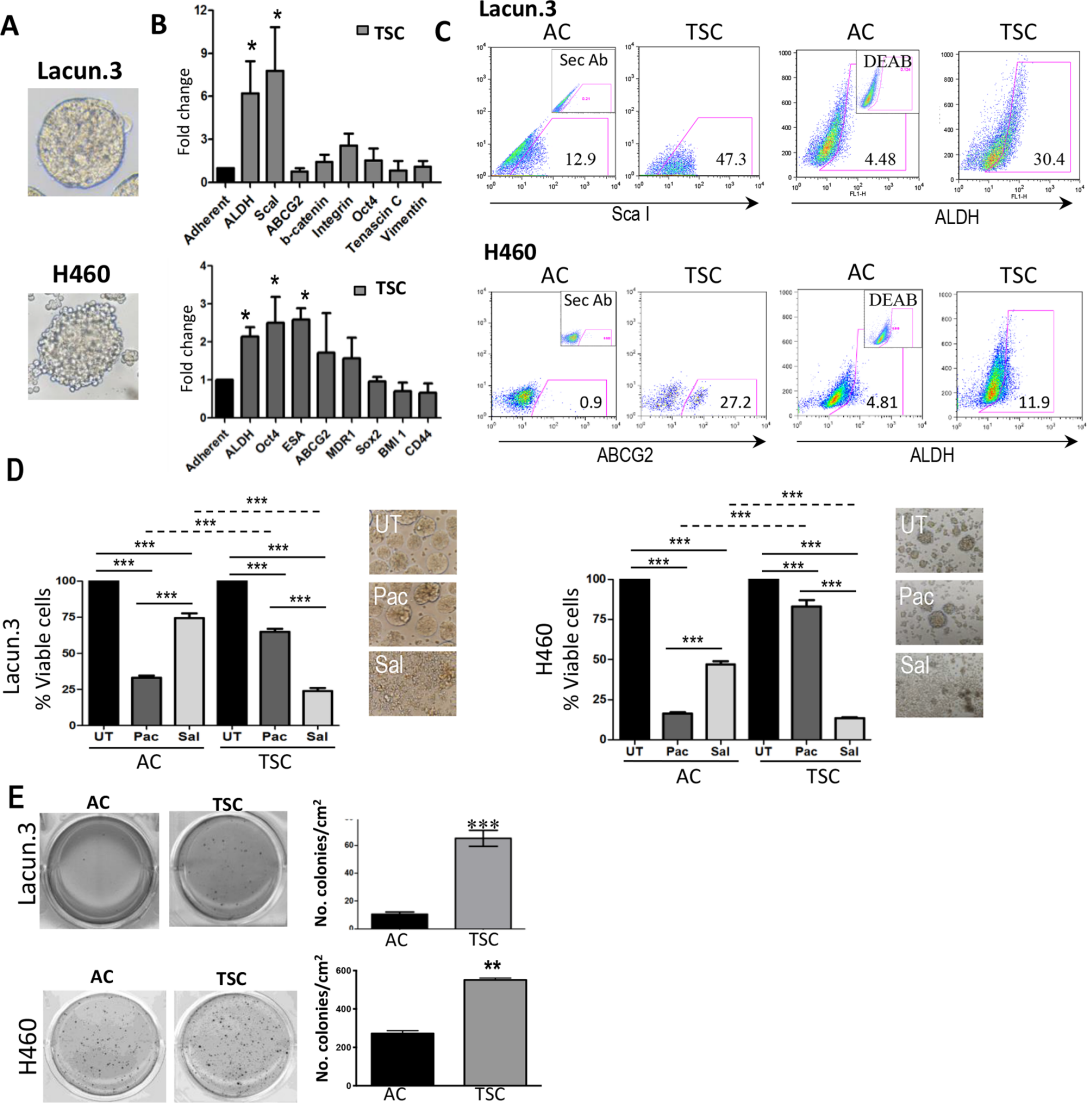
Tumor sphere cultured (TSC) cells exhibit a cancer stem-like cell phenotype and chemoresistance **A.** Representative images obtained from primary lung adenocarcinoma murine Lacun.3 cells and human lung adenocarcinoma cell line H460 cultured under TSC conditions. **B.** RT-qPCR showed greater expression of the stem cell markers ALDH, ScaI in Lacun.3 and ALDH, OCT4 and ESA in H460 TSC versus adherent cultured (AC) cells. **C.** Analysis by flow cytometry showed higher protein expression levels of ScaI, ALDH and ABCG2, ALDH in Lacun.3 and H460 TSC respectively, as compared to AC cells. **D.** *Left panel*: Quantification of cell viability of TSC and AC cells after one week treatment with salinomycin (Sal) and paclitaxel (Pac) (***, *p* < 0.001). *Right panel*: Representative phase-contrast microscopy images of untreated (UT) and treated TSC with Sal and Pac. This experiment was repeated three times with similar results. **E.** Soft agar assay showing an increased anchorage-independent growth ability of TSC versus their matched AC cells. This experiment was repeated three times with similar results. (*p, 0.05; **p < 0.01; ***, *p* < 0.001), Error bars are mean ± SEM.

We prepared TSC from both models. Lacun.3 spheres exhibited delimited spherical structures that could be maintained over multiple generations (Figure 1A and Supplementary Figure S1A). TSC displayed a 6 to 8-fold increase in the mRNA level of the stem cell markers Sca-1 and ALDH, as compared to matched adherent cultures (AC) (Figure 1B, upper panel) (*p* < 0.05). These changes were associated with higher cell surface expression of Sca-1 protein and greater ALDH activity by flow cytometry analysis (Figure 1C upper panel, quantification in Supplementary Figure S1B, *p* < 0.01). In the case of H460 cell line, spheres presented a more irregular shape as compared to Lacun.3 cells but could also be maintained during several passages (Figure 1A lower panel and Supplementary Figure S1A). As compared to AC, TSC showed a significant increase in the expression of various stem cell markers such as ALDH, Oct4 and ESA (epithelial specific antigen) (Figure 1B lower panel) (*p* < 0.05). A tendency for higher ABCG2 level was also detected, although there was inter-experimental variability. Nonetheless, a consistent increase in ABCG2 staining and ALDH activity were detected by flow cytometry (Figure 1B lower panel and Supplementary Figure S1B). These data indicate that TSC overexpress some markers associated with the acquisition of stem cell-like phenotype as compared to cells cultured under adherent conditions.

### TSC cells exhibit lower proliferation rate and are resistant to conventional chemotherapy

Next, we assessed the growth kinetics of TSC and AC cells in the presence or absence of paclitaxel, a first line treatment in lung cancer patients. We found a dramatic decrease in cell growth for Lacun.3 TSC as compared to AC cells, reaching a 15-fold reduction at day 4 (Supplementary Figure S1C) (*p* < 0.001). Paclitaxel strongly reduced the proliferation of AC cells, whereas sphere cell growth was merely affected: sensitivity was 35% greater in the case of AC (Supplementary Figure S1C) (*p* < 0.001). Similar results were obtained for the human H460 cell line. TSC harbored a 5-fold reduction in *in vitro* growth kinetics and a greater resistance to paclitaxel than AC, reaching up to 65% (Supplementary Figure S1C) (*p* < 0.001).

Salinomycin was identified in a drug screening assay to specifically eradicate CSCs [41]. To better document the stem properties of the cells grown in sphere conditions, we measured the impact of this compound on our cultures in parallel with paclitaxel. After 7 days of treatment, salinomycin profoundly disturbed the growth of TSC, producing a 76% reduction in viability (Figure 1D) (*p* < 0.001). Of particular note, treated cultures presented an appearance of disaggregated spheres (Figure 1D). An opposite pattern was found for the treatment of AC cells, which presented higher sensitivity to paclitaxel than to salinomycin, of about 40% (Figure 1D) (*p* < 0.001). Similar results were obtained with H460 cells. Consistently, salinomycin yielded strong cytotoxic effect on spheres, leaving only 13% of cell viability at the end of the experimental period, while producing small effect on AC (Figure 1D, right panel) (*p* < 0.001). Overall, these data underline the different growth properties of TSC and AC cells, and confirmed the sensitivity of TSC cells to salinomycin, a drug typically targeting cells displaying a CSC phenotype.

### TSC showed a higher tumorigenic potential but mitigated metastatic activity

Consistent with their ability to grow under anchorage independent conditions, TSC harbored higher colony formation activity in soft agar assay, reaching up to 6-fold increase in Lacun.3 cells and 2-fold in H460 cells (Figure 1E) (*p* < 0.01). Such trait for a transformed cell reflects a greater *in vitro* tumorigenic capacity.

**Figure 2:**
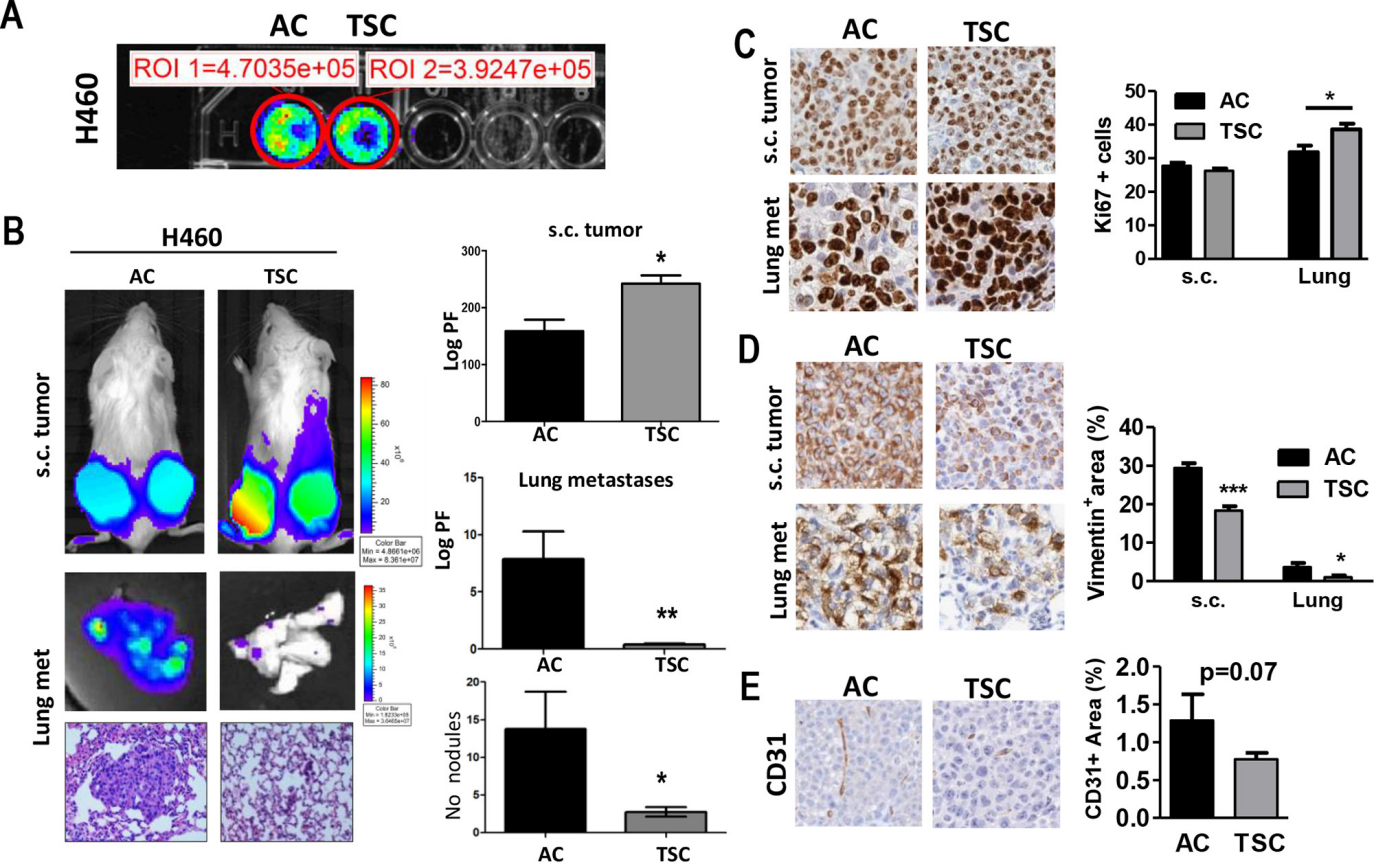
TSC cells display higher tumorogenic potential and decreased metastatic activity **A.** *In vitro* BLI of adherent cultured (AC) cells and disaggregated tumor sphere cultured (TSC) cells for H460 cell line (20 000 cells per well). **B.** *Left panels*: Representative images of BLI of total body (*upper panel*) and excised lungs upon necropsy, and representative H&E stained lung sections for both groups (*bottom panel*). Two groups of mice were subcutaneously (s.c.) injected with H460 cells (TSC and AC). *Right panels*: Quantification of BLI (Photon flux: Log PF) showing increased tumorigenic potential of TSC cells after subcutaneous injection into mice (*upper panel*), while AC cells led to an increased lung tumor burden (*middle panel*) and the number of lung nodules (*lower panel*) in lung histological sections. Error bars are mean ± SEM. (*p* < 0.05). **C.** Representative IHC for Ki67^+^ in resected s.c. tumors and lung metastases in TSC versus AC-injected mice. *Right panel*: Quantification of proliferating cells in both s.c. tumors and lung metastases. **D.** Representative vimentin staining in s.c. tumors (*left panel*) and quantification (*right panel*) for both s.c. tumors and lung metastases, showing reduced positive area in TSC-derived tumors. **E.** IHC staining and quantification for CD31^+^ in s.c. tumors showing a trend towards a decrease in angiogenesis but it did not reach statistical significance. *p, 0.05; **p < 0.01; ***, *p* < 0.001), Error bars are mean ± SEM.

Tumor-initiating ability is a key hallmark for CSCs. We next tested the *in vivo* tumorigenic potential of TSC after subcutaneous injection into athymic nude mice. Before the injection, a comparable luciferase signal was detected *in vitro* for both cell groups (Figure 2A). We found a significant increase in primary tumor growth upon injection of H460 cells derived from disaggregated TSC cells as compared to AC cells (*p* < 0.05). Representative bioluminescence imaging (BLI) and quantification 4 weeks post-injection showed an increase in subcutaneous tumor growth in cells derived from TSC conditions as compared to AC cultures (Figure 2B). Since these tumors can lead to spontaneous lung metastasis, we evaluated BLI in resected tissues. Intriguingly, the appearance of spontaneous lung metastases was reduced in the lungs of animals injected with TSC as compared to AC (Figure 2B, *p* < 0.05). Interestingly, Ki67 staining in resected s.c. tumors demonstrated similar proliferation index for both conditions measured at the end of the experimental period (Figure 2C). In contrast, lung metastases exhibited a higher proliferation index in TSC derived metastases despite a lower size, as compared to AC derived metastases at the end of the experimental period (Figure 2C). However no changes in active capase-3 immunostaining were detected in both subcutaneous tumors and lung metastases (data not shown). Of particular note, vimentin, an intermediate filament associated with a mesenchymal phenotype, was found strongly increased in both primary and lung tumors that originated from AC as compared to TSC derived tumors (Figure 2D).

The same observation was further validated using the Lacun.3 cell line, where TSC produced larger primary tumor volume (*p* < 0.001), but lower metastatic burden in the lungs (*p* < 0.05) (Supplementary Figure S2, *p* < 0.05). In the same line, we observed a trend towards a higher CD31+ immunostaining in subcutaneous tumors derived from AC cells, indicating a tendency for higher vascularization, although it did not reach statistical significance (Figure 2E) (*p* = 0.07). This parameter could not be evaluated in lung metastases because of the small size nodules induced in TSC injected mice.

Finally, ALDH^+^ immunostaining was lower in TSC derived tumors and lung nodules in comparison with AC derived lesions indicating that the stem cell population derived from TSC was not enriched in this marker at the end of the experimental period. The ALDH+ expression was rather uniform between subcutaneous and lung lesions for AC and TSC derived cells (Supplementary Figure S3).

### TSC harbor a lower metastatic activity than AC cultures

**Figure 3:**
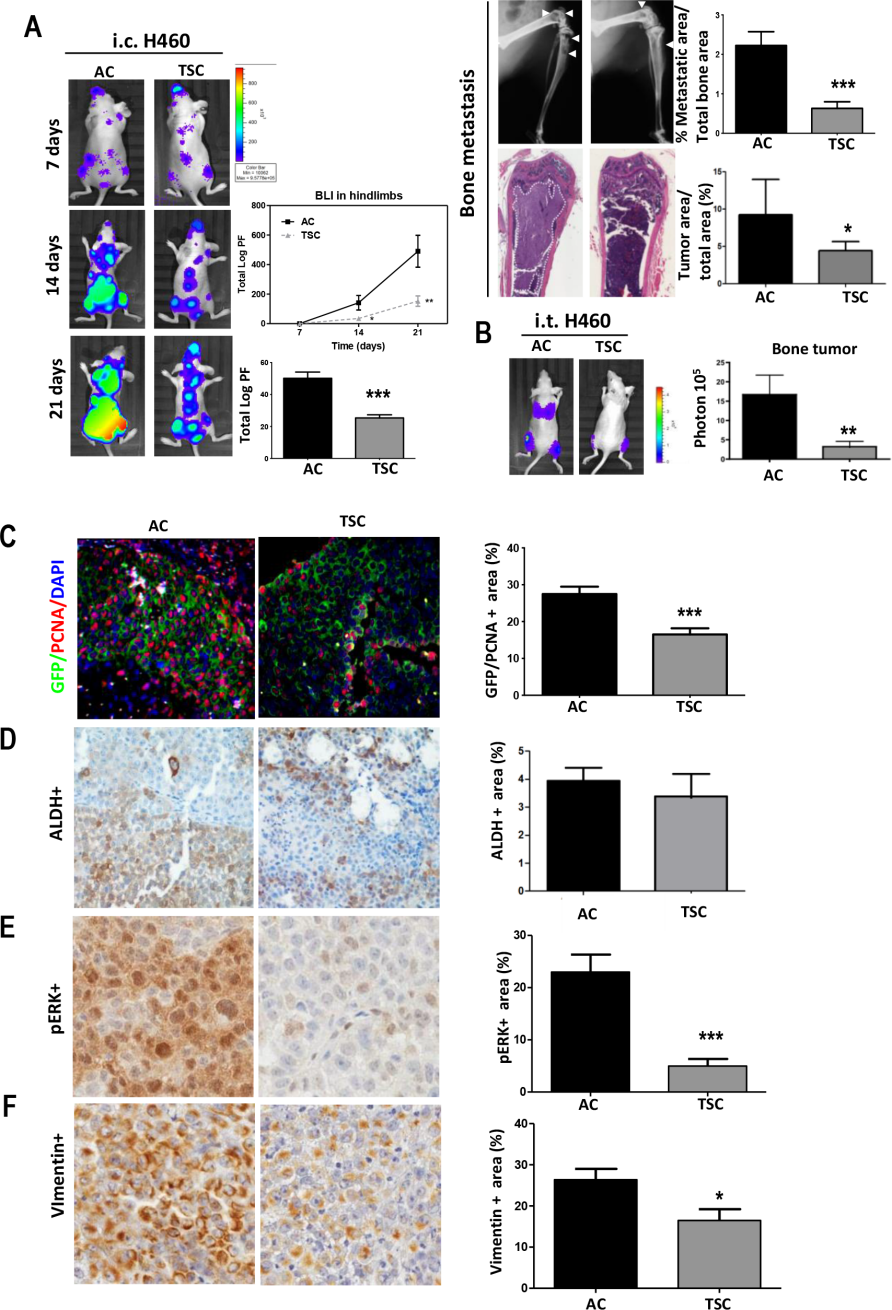
Decreased prometastatic activity of cells derived from TSC conditions **A.** *Left panels*: Representative images of BLI in mice after intracardiac inoculation (i.c.) of H460 AC or TSC cells in two groups athymic nude mice, demonstrating reduced bone metastasis formation in the hindlimbs and whole body. Quantification of total BLI (Photon flux: Log PF) in hind limbs (*upper panel*) and whole body (*lower panel*). *Right panels*: Representative images of X-Ray and H&E. Osteolytic area of the hind limbs (*upper panel*) and tumor area (*lower panel*) for each group are shown. White arrows point to the osteolytic lesions. Quantification of osteolytic area in X-ray images (*upper panel)* and tumor metastatic area (*lower panel*) are shown. **B.** Intratibial injection of H460 cells (TSC and AC cells) in two groups of athymic nude mice followed by BLI measurement and quantification demonstrated reduced osseous colonization in TSC-injected mice. **C.** Double immunofluorescence and quantification for tumor cells (GFP, green) and proliferative cells (PCNA, red) in bone metastasis after i.c. inoculation of H460 cells, showing lower proliferation in TSC-injected mice (at day 21). **D.** IHC and quantification of positive tumor area for ALDH, **E.** pERK and **F.** Vimentin. Error bars are mean ± SEM. *p, 0.05; **p < 0.01; ***, *p* < 0.001.

These unexpected results led us to further investigate the metastatic capacity of TSC cells using another model of metastasis by intracardiac inoculation (i.c.), which recapitulates later stages of metastasis, including cell survival in the circulation, homing and colonization of the target organ [42]. Surprisingly, we found a striking lower metastatic potential upon injection of H460 TSC as compared to AC cells. Of note, tumor cells reached the bone compartment and other sites at day 7. Representative images of BLI, X-Ray and H&E for each group are displayed in Figure 3A. Quantification of BLI in the hind limbs during the experimental period showed a marked decrease in BLI that was further exacerbated from day 14 to day 21 postinjection (*p* < 0.01). A whole body BLI quantification showed 5-fold decrease in total luciferase signal in mice injected with TSC as compared to mice injected with AC cells (Figure 3A) (*p* < 0.001). Osseous metastasis assessed by X-ray image analysis showed proportional reduction of osteolytic lesions in the hindlimbs (Figure 3A) (*p* < 0.01). Histological analysis revealed reduced tumor area vs. the total bone area ratio in TSC and AC-inoculated mice (Figure 3A). As additional control, we used sphere cultures that were disaggregated and plated in AC conditions for 24–48 h before the injection, at equal confluence than AC cells. We found a striking lower metastatic potential upon injection of H460 TSC and plated TSC cells as compared to AC cells (Supplementary Figure S4A).

Analogous observations were made for the Lacun.3 cell line (Supplementary Figure S4B). The i.c. inoculation of TSC cells led to substantially lower metastasis activity than that of AC cells, as shown by significant reduction in total BLI signal at day 21 (*p* < 0.01). X-ray quantification and histological analysis revealed a proportional decrease in osteolytic lesions and tumor burden (Supplementary Figure S4B) (*p* < 0.001). Early time point BLI (7 days) of TSC-injected mice confirmed that the cells reached the target organ (not shown). As an additional group to control for different culture media used between TSC and AC, we cultured disaggregated TSC cells for 24–48 hours in AC conditions before injection (plated spheres). As expected plated spheres behaved like TSC cells (Supplementary Figure S4C).

Similar delay in metastasis development was encountered in TSC cells derived from the A549 cell line. Intracardiac inoculation (i.c.) of TSC cells led to significant decrease in total BLI signal and osteolytic lesions as compared to AC cells (Supplementary Figure S5A, S5B).

To further substantiate whether these observations in lung cancer-derived cells, we prepared TSC from a cancer patient with a malignant pleural effusion (cells were named Mai9) as previously described [43]. The spheres presented strong increase in ALDH, Sox2 and Oct4 mRNA levels as compared to AC (Supplementary Figure S6A, B). As previously shown for other cells lines, TSC displayed a delayed metastatic activity as compared to AC (Supplementary Figure S6C) (*p* < 0.001). These data indicate that TSC cells show a diminished ability to efficiently initiate bone metastatic lesions as compared to AC cells.

### TSC have reduced bone colonization ability

We next evaluated osseous colonization activity by intratibial injection. Consistent with previous results, we found a 6-fold decrease in bone tumor burden upon injection of TSC in comparison with AC, as reflected by lower BLI and tumor area (Figure 3B). Interestingly, as soon as 7 days post-injection, mice injected with AC cells presented prominent signal assessed by BLI in the lungs, which was absent in mice injected with TSC cells (Figure 3B) (*p* < 0.01). Similar results were obtained using Lacun.3 cells (Supplementary Figure S7A). The absence of lung metastasis might have resulted from the lower bone colonization ability of TSC: a proportional decrease in lung BLI, tumor area and number of nodules was detected (Supplementary Figure S7B) (*p* < 0.01).

### TSC cells remain in a quiescent state in the bone

It is worthy to note that after i.c. inoculation of TSC cells, all mice eventually developed metastasis, suggesting that the cells isolated from TSC reached efficiently the distant sites. This could be appreciated in mice i.c. injected with Lacun.3 spheres that were kept up to 34 days (Supplementary Figure S8A). Similar observation was performed in mice injected with H460 TSC at day 21 (Supplementary Figure S8B). Consequently, the decrease in metastatic potential may result, at least in part, from a delay in the ability to initiate efficient colonization. Based on the lower *in vitro* proliferation rate of TSC, we performed double immunohistochemistry for PCNA and GFP in bone sections from mice i.c. inoculated with H460 cells (at day 21). Quantification of double positive cells for PCNA and GFP showed higher number of tumor proliferating cells in mice injected with AC cells than in mice injected with TSC cells (Figure 3C) (*p* < 0.001). On the contrary, TSC-inoculated mice presented numerous resting tumor cells (GFP-positive and PCNA-negative). Staining for ALDH did not show any difference in the percentage of positive cells within the bone metastasis, suggesting that the stem cell population was not enriched in this marker at this time point (Figure 3D). A quiescent state at metastatic site was further evidenced by reduced pERK positive area in mice injected with TSC as compared to AC (Figure 3E). Finally, lower level of vimentin was detected in tissues derived from TSC, suggesting a loss of a mesenchymal phenotype (Figure 3F).

### TSC cells present a quiescent phenotype

**Figure 4:**
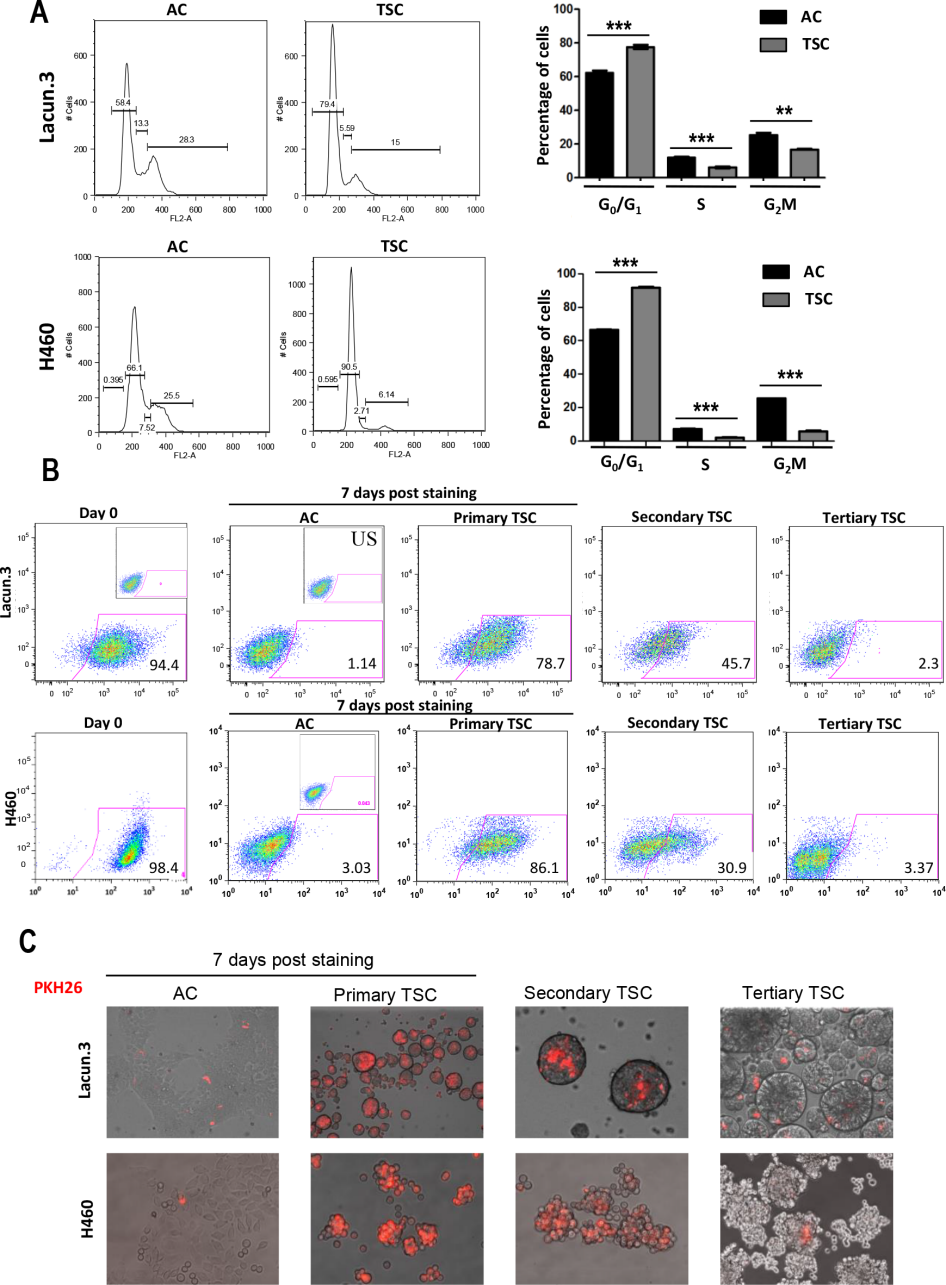
*In vitro* quiescent phenotype in cells cultured in TSC conditions **A.** *Left panel*: Cell cycle distribution analysis after propidium iodide staining by flow cytometry. *Right panel*: quantification of the percentage of cells in each phase. Cell cycle distribution analysis and Lacun.3 cells underlined a cell cycle arrest of spheres in the G_0_/G_1_ phase of the cell cycle. **B.** 7 days post-staining with PKH26, spheres presented greater dye retention ability as compared to AC cells (78% versus 1% of positive cells for Lacun.3 and 86% versus 3% of positive cells for H460), as shown by flow cytometry analysis. **C.** Representative images of fluorescent microscopy showing dye dilution upon secondary and tertiary sphere generation. All experiments were repeated at least three times with similar results.

The lower proliferation rate of TSC cells observed *in vitro* and *in vivo* led us to evaluate cellular quiescence. We first assessed the cell cycle distribution of cultured cells. For both Lacun.3 and H460 TSC, we found a marked cell cycle arrest in the G_0_/G_1_ phase of about 20% (Figure 4A) (*p* < 0.001) as compared to AC cells. This was accompanied by a lower percentage of cells in the S and G_2_M phases in both cell lines (Figure 4B) (*p* < 0.001). BrdU assays confirmed this trend showing a 7-fold reduction in the percentage of cells in S phase as well as an increase in G_0_/G_1_ phase in H460 TSC when compared with H460 AC cells (Supplementary Figure S9). We then used a fluorescent-dye retention assay to further address cellular quiescence. The PKH26 dye, which becomes diluted after each cell division, has been previously shown to accumulate into stem cells [44]. One week after initial staining, TSC derived from Lacun.3 cells displayed 80% higher PKH26 staining than AC cells, as shown by flow cytometry analysis (Figure 4B). The PKH26 signal was then reduced upon secondary and tertiary sphere generation, leaving a remaining ~2% population of positive cells (Figure 4B). Representative images of spheres stained with PKH26 are displayed in Figure 4C. Comparable data were obtained for the H460 cells, where TSC displayed higher ability to retain the PKH26 dye than AC cells, as shown by flow cytometry analysis and fluorescent microscopy imaging (Figure 4B and 4C). These results indicate a higher number of quiescent slow cycling cells in TSC than in AC cells.

### Altered quiescence signaling pathways in TSC

**Figure 5:**
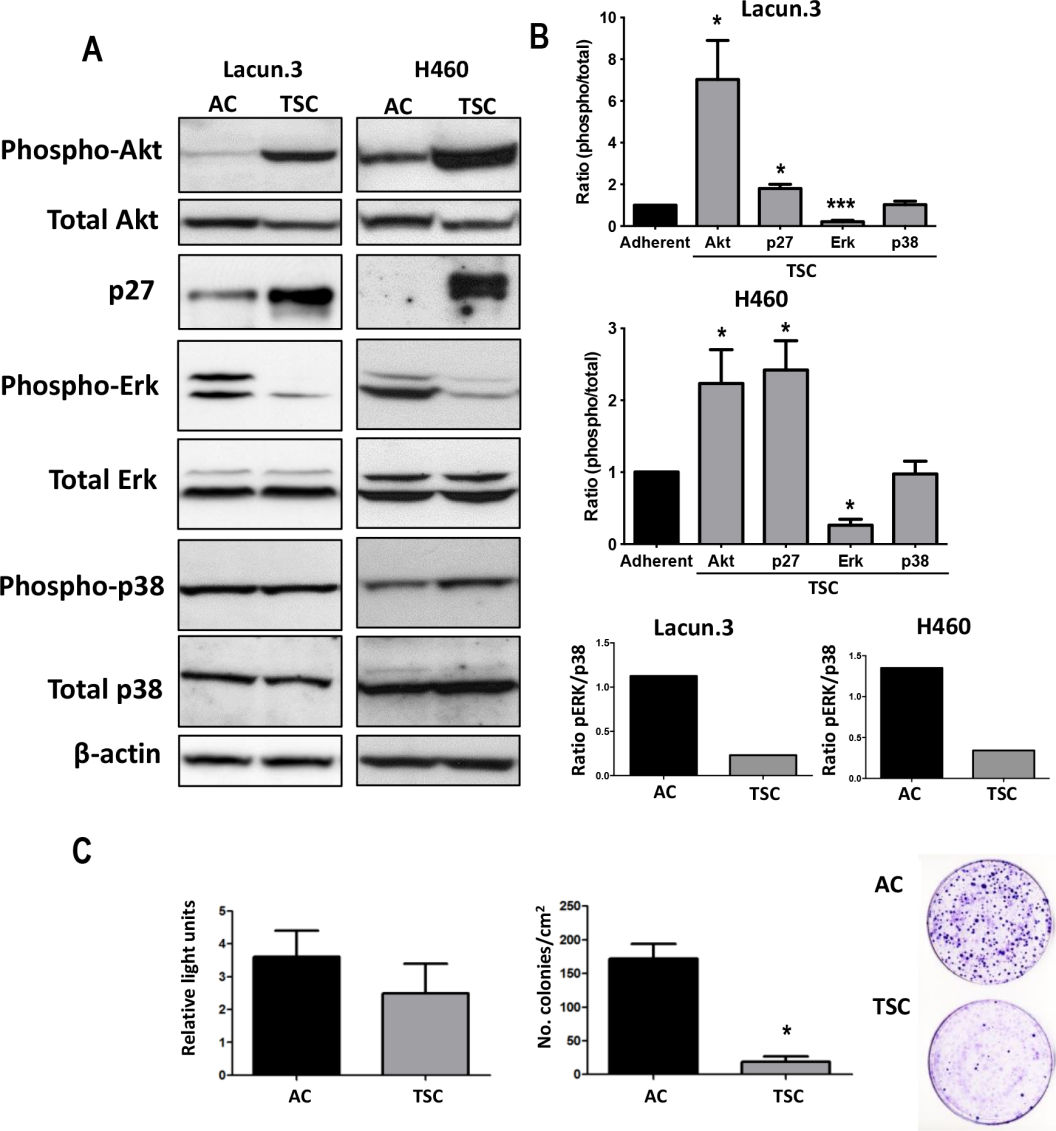
TSC cells express markers of quiescence *in vivo* **A.** Representative immunoblots of cell extracts obtained from *in vitro* cultures showing strong increase in the active phosphorylated form of Akt in TSC cells as compared to AC cells. A decrease in phospho-Erk was detected, but with similar levels of phospho-p38. Normalization was obtained by detection of all forms (total) and β-actin. TSC cells presented higher levels of p27 protein than AC cells. Experiment was repeated 3 times with similar results. **B.** Fold change in densitometric values for AC cells as compared to TSC cells from Lacun.3 cells (*Top*) and H460 cells (*Middle*). (*Bottom)* Fold change in densitometric values for AC and TSC cells showing a decreased ratio of phospho Erk/phospho-p38. Ratios correspond to the measurement of phosphorylated proteins on total protein or actin. (*, *p* < 0.05; ***, *p* < 0.001). Error bars represent mean ± SEM. **C.** *Left panel*: Two groups of mice (8 animals per group) were i.c. inoculated with TSC and AC cells. Animals were sacrificed 6 days post-inoculation and tibiae were excised and cells were extracted by bone-marrow flushing and cultured in neomycin medium until the emergence of single-cell derived colonies. Relative light units of BLI in mice injected with AC cells and TSC cells from the H460 cell line at day 6. *Left panels*: Number single-cell derived colonies obtained after bone marrow “flushing” and grown during 10 days in regular medium.

We then investigated the modulation of different signaling pathways related to cellular proliferation. We found a strong increase in the level of pAkt in TSC isolated from both Lacun.3 and H460 cells as compared to AC cells (*p* < 0.05) (Figure 5A and 5B). Interestingly, phosphorylation of Akt has been previously shown to associate with the self-renewal ability of normal and cancer stem cells [45], as well as resistance to therapy [46]. TSC cells also presented high levels of p27 protein (*p* < 0.05), a marker of cell cycle arrest and cellular quiescence [28]. We detected a strong decrease in pErk for TSC cells, indicating slower proliferation rate (*p* < 0.001). The levels of p38 were not affected at this time point, however the ratio of pErk/p38 for TSC cells was reduced as compared to AC cells in both cell lines (Figure 5A and 5B). A low ratio pErk/p38 has been associated with cancer cell dormancy [47]. Overall these results indicate that a quiescent phenotype could be involved in the delayed prometastatic activity observed in TSC *in vivo*.

### TSC display a quiescent *in vivo* metastatic phenotype

To determine whether the delay in colonization observed in TSC-injected mice could result from a difference in homing ability, mice (8 mice per group) were i.c. inoculated with cells and sacrificed 6 days later. At this time point, tumor circulating cells have efficiently reached the target organ. In this experimental system, we did not find significant change in total luciferase signal upon injection of AC or TSC cells, indicating that a similar amount of cells reached the remote organs (Figure 5C). However, after bone marrow flushing, we found dramatic lower number of single-cell derived colonies (>100 cells) cells isolated from TSC-inoculated mice, suggesting lower growth ability of TSC cells to induce robust colony formation as compared to AC cells (Figure 5C). Taken together these data indicate that TSC cells maintain their quiescent phenotype in the target organ.

## DISCUSSION

In this report, we showed that lung tumor cells selected for prominent cancer stem cell-like properties including enhanced ability to sustain tumor growth and increased chemoresistance, paradoxically exhibit an impaired metastatic activity. This observation was consistently detected in different target organs (lung and bone) and in several human and murine models of metastasis (upon intracardiac inoculation, subcutaneous and intratibial injections). This metastatic pattern was mediated by ERK1/2, p38 and Akt pathways leading to a marked metastatic quiescent phenotype as a consequence of an arrest in G_0_/G_1_ phase in target organs. This event was consistent with the tumor cell-autonomous expression of p27 and a reduced pERK1/2 /phospho-p38 ratio, a mechanistic indication of cell cycle arrest and cellular quiescence at metastatic sites [48]. This finding demarcates distinct and independent tumor-initiating properties (tumor growth initiation and sustainment) displayed by TSC-derived CSC from their prometastatic activity such as their ability to initiate robust colonization at distant sites, an effect heavily influenced by the hosting milieu. Remarkably, cells exhibit robust tumor initiating properties *in situ* when subcutaneously injected whereas a tumor-quiescent phenotype observed *in vitro* in TSC cells was maintained in the lung and in the osseous compartment, leading to decreased colonization after both, intratibial or intracardiac injections. These findings indicate that cell cycle arrest was highly dependent on microenvironmental cues. Previous studies have suggested that metastasis-initiating cells need to overcome organ-specific anti-metastatic signals in order to undergo reactivation [49]. A full engagement with ECM components has been shown necessary for tumor cells to escape from tumor quiescence [50, 51]. Such process involves in part the production of collagen type I and fibronectin, as well as the activation of β1 integrin and ERK signaling to form a permissive niche and turn on cell proliferation [52, 53]. In bone, secretion of osteopontin (OPN), and the soluble vascular cell adhesion protein 1 (VCAM-1) were both reported to tightly regulate cell dormancy [54, 55]. Similarly, bone-derived BMP-7 induced growth-arrest of cancer stem cells which eventually led to tumor dormancy [56]. Overall, ECM proteins involved in tumor-matrix interactions and secreted growth factors in each organ are critical for modulating cell quiescence.

More importantly our work extends the finding of a quiescent state to cells previously selected for their prominent cancer-stem cell phenotype, an effect probably exerted by signaling pathway engagement by these organs. Similar cellular quiescence has been associated *in vivo* with the dormant phenotype of solitary tumor cells encountered in the target organs after dissemination from the primary tumor site [48, 57]. This dormant state was organ-dependent, since the bone marrow and lung impose a restrictive (bone marrow) and permissive (lung) microenvironment for metastasis in a model of a head and neck squamous cell carcinoma [58]. This finding underscores a non-cell autonomous effect in CSCs mediated by the microenvironment despite their robust tumor initiating activity observed in the subcutaneous milieu.

Interestingly, CSC properties displayed by TSC were enriched using sphere culture conditions, a method commonly used to derive CSCs from bulk tumor specimens. Rather than spurious effects triggered by culture conditions, the enrichment in CSC properties was supported by the finding of a similar metastatic phenotype observed in an additional control group of disaggregated TSCs subsequently cultured in AC conditions for 24–48 hours (Supplementary Figure S4B, S4C). In agreement with our findings, TSC isolated from a breast cancer model were shown to express high levels of the stem cell marker Sca-1 and to display a near complete loss of spontaneous metastasis [59], an effect associated with decreased expression of TGFβ2 and reduced activation of the TGFβ signaling pathway in spheres [59]. Thus, differences in the methods employed to isolate and enrich for CSCs could dramatically affect the co-selection of different expression patterns of ECM binding proteins mediating different responses to the constraints imposed at the target sites, resulting in dramatic differences in metastatic properties. In this line, the finding that TSC-derived tumors express less vimentin is consistent with their low prometastatic activity, since gain in vimentin has been shown to increase filopodia, invasiveness and metastasis [60]. Thus, the TSC isolation method present dissimilarities with other methods commonly used to enrich for CSCs, such as sorting for surface markers. For instance, slightly different medium conditions severely impaired growth of sphere derived H460 cells in subcutaneous xenografts [61], an observation that could be explained by a high CD44 phenotype, unchanged in our case. In contrast to our findings, cisplatin-resistant H460 cells grown in TSC conditions were enriched in CSCs markers (CD133+) and displayed higher tumorigenic and metastatic potential [62], an effect that could be explained by the co-selection of prometastatic properties induced by chemoresistance. Of note, slightly different stem cell markers were enriched in different cell types using our sphere culture conditions (Figure 1 and Supplementary Figure S6). This divergence observed between studies suggests that a main CSC phenotype acquired through different experimental approaches could differentially co-select for prometastatic and/or chemoresistant properties.

Similarly, a subset of CD26^+^ expressing CSC in colorectal cancer endowed with tumor-initiating capacity also showed high metastatic properties to the liver [63]. Alternatively, these discrepancies could be partially reconciled by the possibility that our isolated TSC cells could represent a heterogeneous population containing quiescent and a small fraction of non-quiescent cells more prone to form metastasis.

Nevertheless, our results are consistent with previous reports that used distinct CSC markers [31, 35, 36]. Tumor spheres formed by sorted CD133^+^cells contained more quiescent cells than those derived from CD133^−^cells. Moreover, the established tumor spheres from CD133^+^ cells grown in 3D culture without serum remained quiescent, whereas they exited from a quiescent state after serum addition [64]. These data suggest that TSC contains a pool of CSCs in a predominant quiescent state with different sensitivities to reactivation by microenvironmental cues. Similarly, label-retaining cells (LRC) with self-renewal ability have been identified in a sub-population of CD44+/CD24-/ESA+ breast cancer cells, and these cells were endowed with resistance to chemotherapy [65]. Interestingly, the presence of LCRs has been shown to result from slow cycling cells as well as cells undergoing asymmetric cell division, leading to increased tumor-initiating ability [66, 67].

Of interest, it has been suggested that a migratory EMT cell phenotype must first revert to an epithelial phenotype in order to trigger colonization at distant sites, which is made possible by a mesenchymal-to-epithelial transition (MET) [68, 69]. However, in order to escape from the primary tumor and thrive in the target metastatic organ, different studies have shown that displaying a mesenchymal phenotype confers metastatic advantage [70]. In our study, we have found that both AC-derived primary tumors and metastatic lesions (including both lung and bone), express significantly higher levels of the EMT marker vimentin, associated with increased invasiveness and metastasis. In contrast, in a recent study, more mesenchymal-like prostate cells were less prone to initiate metastatic lesions [71]. Thus, different models exhibit different features (EMT and MET) that differ across tumors or even across different tumor subtypes. Alternatively, cell plasticity allowing EMT/MET might emerge independently of an intrinsic and/or a host-mediated prometastatic phenotype. This plasticity has also been invoked to explain the enrichment in ALDH+ cells observed in xenograft models by AC cells and its expression in primary and metastatic lesions [72], a finding also detected in our experiments (See Supplementary Figure S3).

Several consequences derived from our study could be useful in the clinical setting. For instance, anti-cancer stem cell therapies could be insufficient to eradicate disseminated tumor cells, since other tumor cells with non-cancer stem cell properties could emerge with enhanced metastatic activity. Our observations might also help to explain how low-growth rate tumors at the primary site might display a marked metastatic activity, a recurrent observation made in a fraction of lung adenocarcinoma tumors, which are frequently diagnosed by the detection of overt metastasis in the skeleton [73, 74]. However, our findings need to be cautiously interpreted and extended to other tumors and models.

In summary, the current study indicates that CSC phenotype derived from TSCs shows a reduced metastatic potential as compared to non-TSC, underlined by a delay in colonization of target organs, an effect that is heavily influenced by the host microenvironment. Mechanisms included a quiescent *in vivo* phenotype of TSC at metastatic sites, mediated by a reduction in pERK1/2 /p38 ratio and increase p27 levels. These findings could partially explain several clinical observations regarding metastatic pattern and might have therapeutic implications for the management of secondary outgrowths.

## MATERIALS AND METHODS

### Ethics statement

Investigation has been conducted in accordance with the ethical standards, according to the Declaration of Helsinki, to national and international guidelines and has been approved by the authors' institutional review board.

### Monolayer and tumor spheres cultures

The mouse lung adenocarcinoma cell line, Lacun.3, was isolated and characterized at the University of Navarra (Pamplona, Spain) from a combined silica and N-Nitrosodimethylamine (NDMA)-induced lung cancer model [39]. The human cell line Mai9 was also isolated and characterized by our group from a malignant pleural effusion (MPE) of a patient diagnosed with NSCLC [43]. Lung adenocarcinoma cell lines (H460 and A549) were obtained from the American Type Culture Collection (Manassas, VA). All cells were cultured in RPMI 1640 medium (Invitrogen, Carlsbad, CA) supplemented with 10% FBS and antibiotics at 37°C and 5% CO_2_. Cells were retrovirally transduced with a luciferase reporter gene for *in vivo* bioluminescence image analysis.

To obtain sphere cultures, monolayer cells were enzymatically and manually dissociated into a single cell suspension using Trypsin-EDTA (0.125%) followed by passage through a 25-gauge needle. Cells were seeded at 5000 cells/mL into non-adherent PolyHEMA-coated plates (1.2% poly-(2-hydroxyethylmethacrylate)/95% ethanol) (Sigma-Aldrich, St. Louis, MO). Stem cell medium consisted in DMEM/F12 supplemented with B27 (Gibco-Life Technologies, Carlsbad, CA) and MEGM SingleQuots (human epidermal growth factor, insulin, hydrocortisone and GA-100; Lonza, Basel, Switzerland). Tumor sphere cultured (TSC) cells were disaggregated by incubation with the StemPro^®^ Accutase^®^ Cell Dissociation Reagent (Life Technologies) until a single cell suspension was obtained. TSC cells were subcultured every five to seven days for up to five generations.

### Quantitative real-time reverse transcriptase PCR analysis

Purified RNA was obtained with the Qiagen RNA isolation kit (Chatsworth, CA) and cDNA was synthesized with the SuperScript II First-Strand Synthesis System (Invitrogen). The PCR amplification mixture contained cDNA, SYBR Green I Master Mix buffer (Applied Biosystems, Forster City, CA), and forward and reverse primers (20 nM each). Primers for amplification are shown in Supplementary Table S1. Real time PCR was carried out with the 7300 RT-PCR system (Applied Biosystems). Every assay was performed in triplicate and all experiments included analysis of GAPDH mRNA levels as internal control. Relative expression was determined by the Ct method and levels were expressed as percentage relative to the GAPDH mRNA levels.

### Proliferation and soft agar colony formation assay

Cell viability was determined by MTT (3-(4, 5-Dimethylthiazol-2-yl)-2,5-diphenyltetrazolium bromide) assay (Roche, Switzerland). Disaggregated TSC and adherent cultured (AC) cells were seeded in 96-well culture plates (500 per well) in 100 μL of their medium, in the absence or presence of paclitaxel (10 μM) (Sigma-Aldrich, Madrid, Spain). Twenty four, 48, 72 and 96 h later, 10 μL of MTT (2.5 mg/ml) was added and cells were further incubated for 4 h, followed by addition of 100 μL solubilization buffer overnight. To evaluate the sensitivity to salinomycin (Sigma-Aldrich) cells were incubated during one week with or without salinomycin (1 μg/μL). Spectrophotometric absorbance was measured at 570 nm.

Soft agar assay for colony formation was performed by seeding 1 × 10^3^ cells into 0.3% agar containing RPMI and 10% FBS over 2-ml base layers (0.6% agar). After 10 days in culture, the number of colonies was assessed by staining with 500 μL of 10 mg/mL MTT solution. After incubation for 4 hours, 500 μL DMSO were added, the plates were scanned, and the cell colonies were counted.

### Flow cytometry and cell cycle analysis

For immunostaining, dissociated cells were blocked with 1 μg human IgG/1 × 10^5^ cells and incubated 30 min on ice with PE anti-mouse ScaI or PE isotype control (1:200, BD Biosciences, San Diego, CA); APC anti-human ABCG2 or APC isotype control (1:10).

ALDH staining was performed with the ALDEFLUOR kit (Stem Cell Technologies, Canada) according to the manufacturer's instructions. Briefly, cells were suspended at 1 × 10^6^ cells/ml in Aldefluor assay buffer containing ALDH substrate (BAAA, BODIPY^®^ aminoacetaldehyde, 1 mmol/l), with or without the specific ALDH inhibitor diethylaminobenzaldehyde (DEAB) (1 mmol/l) for 30 min. Analysis and sorting were conducted on a FACSAria IIu (BD). Aldefluor and PE were excited at 488 nm by an octagon blue laser, and fluorescence was detected using 530/30 and 675/20 filters, respectively. APC was excited at 633 nm, and emission was at 660 nm. Dead cells were excluded by gating on forward and side scatter and eliminating the 7 AAD-positive population. The data were analyzed by Cell Quest Pro and *FlowJo* (Ashland, OR). For sorting, cells were separated into ALDH-bright and ALDH-low cells according to their level of activity.

For PKH26 staining, 5 × 10^5^ cells/ml cells were labeled with PKH26 for 5 min following manufacturer's instructions (Sigma). Labeled cells were seeded in complete medium (in regular flask) or in stem cell medium (in non-adherent plates). After 7 days, cells were trypsinized, filtered, and analyzed by flow cytometry in the PE channel, or visualized by microscopy. Second and third generation spheres were disaggregated and reanalyzed.

For cell cycle distribution analysis, disaggregated spheres and monolayer cells were fixed in 4% paraformaldehyde for 5 min on ice, immersed in 70% ethanol and kept at −20°C for 30 min or until analysis. Cells were rehydrated in PBS, treated with RNase A (500 μg/ml) (Qiagen, Germany) and stained with propidium iodide (50 μg/ml).

For BrdU assay (BD), disaggregated TSC and adherent cells were plated until attachment and 50 μM BrdU solution was added for 3 h. Cells were then collected and processed according to manufacturer's instructions. FACSCanto cytometer and FlowJo software were used for acquisition and data analysis.

### Immunohistochemistry and immunofluorescence

Tissues were fixed in 10% buffered formalin, embedded in paraffin, and sectioned (5 μm). For histological analysis, slides were stained with Heamatoxylin & Eosin. For IHC, sections were deparaffinized, hydrated and incubated for 10 min with 3.3% H_2_O_2_ in water to block endogenous peroxidase. Standard protocols for antigen retrieval were employed. Dilutions of primary antibodies were as follows: 1:100 for Ki67 (Dako, Denmark), 1:50 for CD31 (Dianova, Germany), 1:500 for Vimentin (Dako), 1:50 for pERK (Cell Signaling, MA, USA), 1:500 for anti-PCNA (Clone PC10, Dako); 1:1000 human anti-ALDH (BD Biosciences) and 1:500 anti-GFP (Abcam, Cambridge, MA). Samples were incubated with primary antibodies at 4°C overnight. For immunohistochemistry detection, slides were incubated for 30 min with the EnVision™ anti-mouse or anti-rabbit detection system (Dako). Peroxidase activity was developed with DAB (3,3′-diaminobenzidine; Dako). Images were captured with a Nikon microscope Y-TSH (Japan). For immunofluorescence, fluorochrome-labeled secondary antibodies conjugated to Alexa-Fluor 488 and 568 (1:500, Invitrogen) were incubated at room temperature for 30 min. Slides were washed and then incubated with 0.1 μg/mL DAPI for 1 min in darkness. Images were captured with a fluorescent microscope (Zeiss AXIO Imager Z1) equipped with Imaging System V.5.0 software (MetaSystem GmbH, Altlussehem, Germany). For quantifications, 30 random images (× 200) per experimental group were used. The stained areas were quantified with the ImageJ software (NIH, Bethesda, MD) and data were expressed as positive immunostained area with respect to reference area.

### Western blot

Proteins were extracted in RIPA buffer and concentrations were measured by the bicinchoninic acid method (Pierce, IL). Total extracts were electrophoretically fractionated on 10% or 12% Bis-Tris polyacrylamide gels (Invitrogen) and transferred to a 0.45 μm nitrocellulose membrane. Membranes were blocked for 1 h with 5% nonfat dry milk in TBS-Tween and incubated overnight at 4°C with the following primary antibodies: phospho-p38, phospho-Akt, phospho-Erk1/2, and total p27, p38, Akt and ERK (diluted 1:1000, Cell Signaling, Danvers, MA), p21 (Dako, 1:1000), and β-actin (Sigma, 1:5000). Membranes were washed and incubated with peroxidase-labeled secondary antibodies at room temperature for 1 h. Immunoreactive bands were detected with the Lumi-Light Western Blotting Kit (Roche, Switzerland) and quantified with ImageJ.

### *In vivo* assays

Female athymic nude mice and Rag2^−/−^/ILR2γ^−/−^ (Harlan Ibérica, Spain) were maintained under specific pathogen-free conditions. All the animals were sacrificed according to the approved protocols of the Local Animal Committee. Cells were previously transduced with a triple modality construct containing a triple fusion protein GFP-luciferase-thymidine kinase [75]. Four-week old nude mice (8 mice/group) were inoculated in the left cardiac ventricle with 2 × 10^5^ cells (monolayer cells or tumor sphere cultured cells from third generation, either freshly disaggregated or plated in regular culture dish 24–48 h prior to injection), in 100 μl of PBS as detailed elsewhere [40, 76]. Subcutaneous experiments were performed on Rag2^−/−^/Il2Rγ^−/−^ mice together with the i.c. inoculation of Lacun3, a fact that was dependent on the availability of mice in the in-house colony.

For bioluminescence imaging and analysis, mice were anaesthetized and injected intraperitoneally with 1.5 mg of D-luciferin in 100 μl of PBS. Imaging was completed at 2 min exactly for each group of mice with a Xenogen IVIS system coupled to Living Image acquisition and analysis software (Xenogen Inc., CA). Photon flux was calculated for each mouse by using a circular region of interest for each hind limb. Background value (from luciferin-injected mouse with no tumor cells) was subtracted from each measurement. X-ray radiography was performed by placing mice in the prone position on sensitive radiographic film (MIN-R, Eastman Kodak). The percentage of osteolytic area of femur and tibia respectively for each animal compared to the total bone area of femur and tibia was assessed with computerized image analysis system, AnalySIS^®^ (soft imaging system GmbH, Münster, Germany). High resolution X-ray film scans with 2 × magnification were captured at 1200 ppi using a Epson Expression 1680 Pro scanner (Long Beach, CA).

Bone colonization ability was evaluated after intratibial injection into nude mice (6 per group). Briefly, cells were suspended at 2 × 10^6^ cells/mL in sterile PBS. Mice were anesthetized before injection. Five microliters containing 1 × 10^4^ cells were injected into the tibia's bone marrow through femoro-tibial cartilage of the 6-week-old mice (Harlan Ibérica) using a Hamilton syringe. For the spontaneous lung metastasis experiment, 5 × 10^4^ cells/mL were injected subcutaneously (8 mice/group). After 5 weeks, mice were sacrificed and the lung analyzed by bioluminescence imaging and H&E. All *in vivo* experiments were normalized on the luciferase signal at day 0.

### Quantification of single cell-derived bone colonies

To evaluate the homing ability of tumor cells at six days post intracardiac inoculation, long bones were excised and cleaned of all soft tissues. Marrow cells were released by “flushing,” introducing 5 to 10 mL of α-MEM medium containing penicillin/ streptomycin with a 27-gauge needle in the distal epiphysis through the bone marrow compartment. Cell clumps were disaggregated by passing medium containing cells through 27-gauge needle syringe. Cells were plated in 10- or 15-cm dishes expanded for 10 days in medium containing 0.5 mg/mL G-418. This procedure was conducted separately for each femur and tibia of 7 mice per group. Single cell-derived colonies (SCDC) were counted under light microscope after crystal violet staining.

### Statistical analysis

To study differences in metastatic area, proliferation rates, analysis of bioluminescence imaging, and gene expression levels, data were analyzed by parametric test or non-parametric homologue Mann-Whitney *U* test depending on data distribution. Values were represented as mean ± SEM. Statistical significance was defined as (*) *p* < 0.05, (**) *p* < 0.01, and (***) *p* < 0.001.

## SUPPLEMENTARY FIGURES AND TABLES



## Abbreviations

TSC: tumor sphere cultures
AC: adherent cultures
BLI: bioluminescence imaging
i.c.: intracardiac
i.t.: intratibial
