# Supplementary material for: Sphere-derived tumor cells exhibit impaired metastasis by a host-mediated quiescent phenotype
Source: Oncotarget. 2015 Aug 7;6(29):27288–303. doi: 10.18632/oncotarget.4803 (PMC4694990; doi:10.18632/oncotarget.4803)
Supplement: Supplementary file 1 [file oncotarget-06-27288-s001.pdf]

SUPPLEMENTARY FIGURES AND TABLES

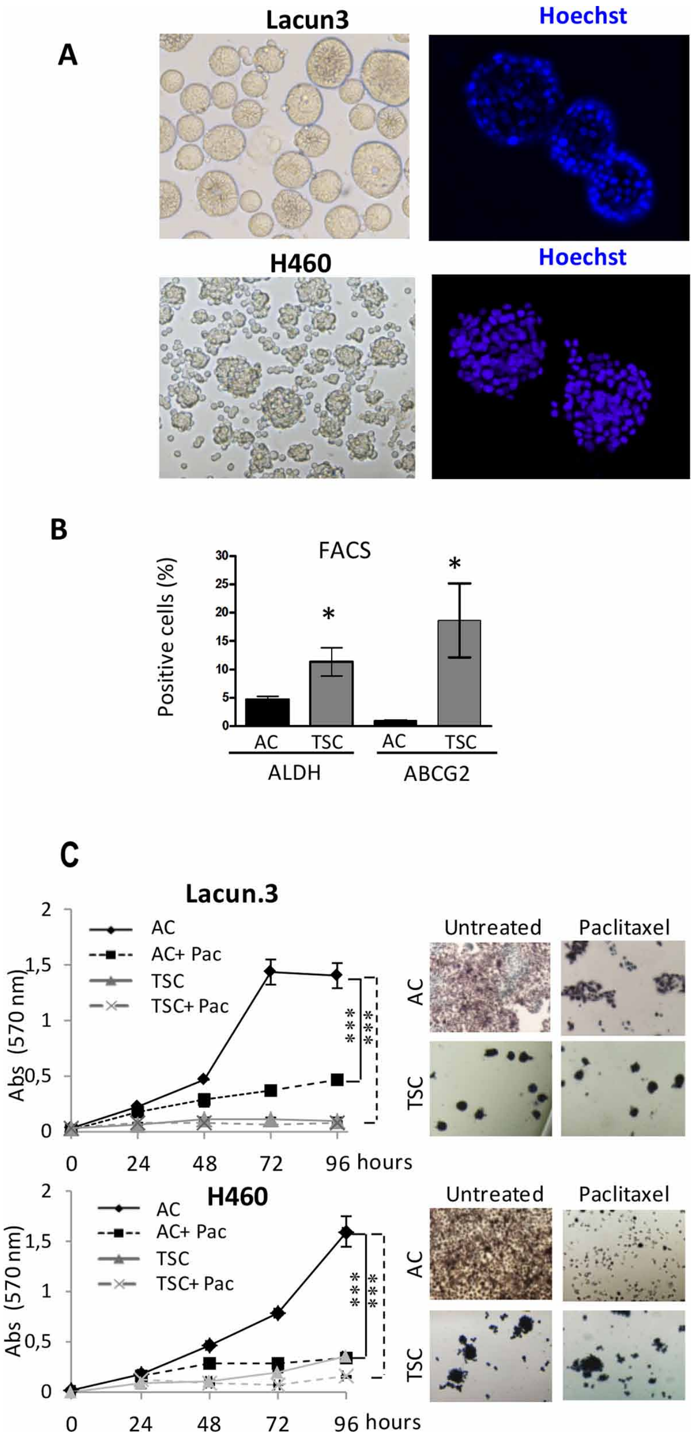

Supplementary Figure S1: Tumor sphere culture (TSC) from Lacun.3 and H460 cells. A. Phase contrast microscopy (*left*) and Hoechst staining (*right*) showing high sphere formation ability of Lacun.3 cells and H460 cells. **B.** Flow cytometry analyses demonstrate higher levels of ALDH and ABCG2 in H460 spheres as compared to adherent cells. Error bars are mean ± SEM. *, $p < 0.05$. **C.** Left panel: *In vitro* proliferation assessed by MTT assay of cells grown in AC and TSC conditions. TSC cells were cultured, disaggregated and seeded into non-adherent 24-well plates. MTT assay on H460 cells demonstrated the lower proliferation rate of TSC as compared to AC (full line; ***, $p < 0.001$). Paclitaxel (Pac) strongly reduced the viability of AC (dashed line; ***, $p < 0.001$). Right panel: Representative phase-contrast microscopy images of cells stained with the MTT substrate.

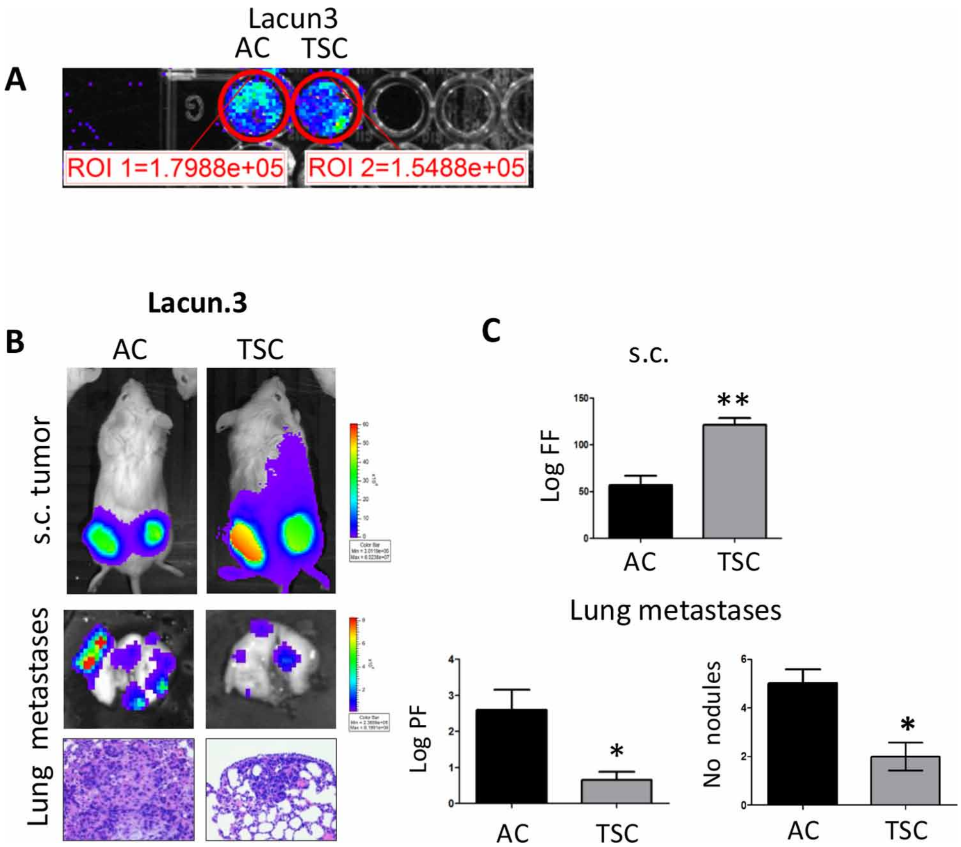

Supplementary Figure S2: TSC present higher tumorigenic potential with decreased metastatic activity versus AC cells. **A.** *In vitro* BLI of adherent cultured (AC) cells and disaggregated tumor sphere cultured (TSC) cells for Lacun.3 (10 000 cells) **B.** Two groups (8 mice/group) of animals were subcutaneously injected with Lacun.3 TSC and AC cells. Representative images of BLI of total body (*upper panel*) and excised lungs upon necropsy, and representative H&E stained lung sections for both groups (*bottom panel*). **C.** Quantification of BLI showing increased tumorigenic potential of Lacun.3 TSC cells after subcutaneous injection into mice. AC cells led to an increased lung tumor burden as shown by BLI and number of nodules.

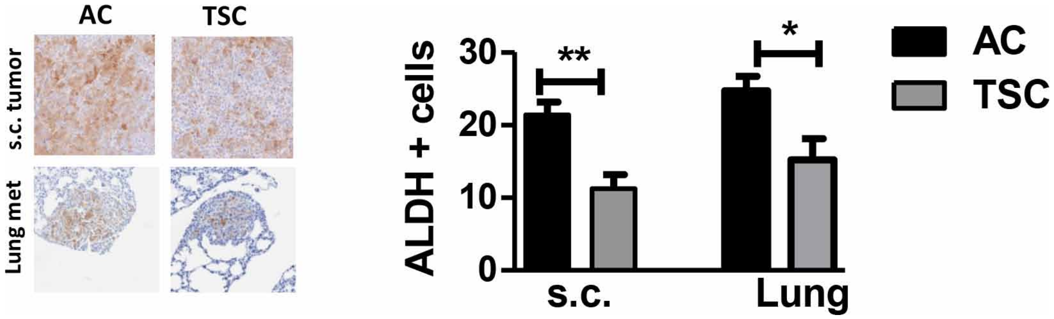

Supplementary Figure S3: ALDH+ immunohistochemistry. Immunohistochemical analysis of ALDH+ cells in subcutaneous tumors and in spontaneous lung metastatic lesions after injection of AC and TSC derived cells.

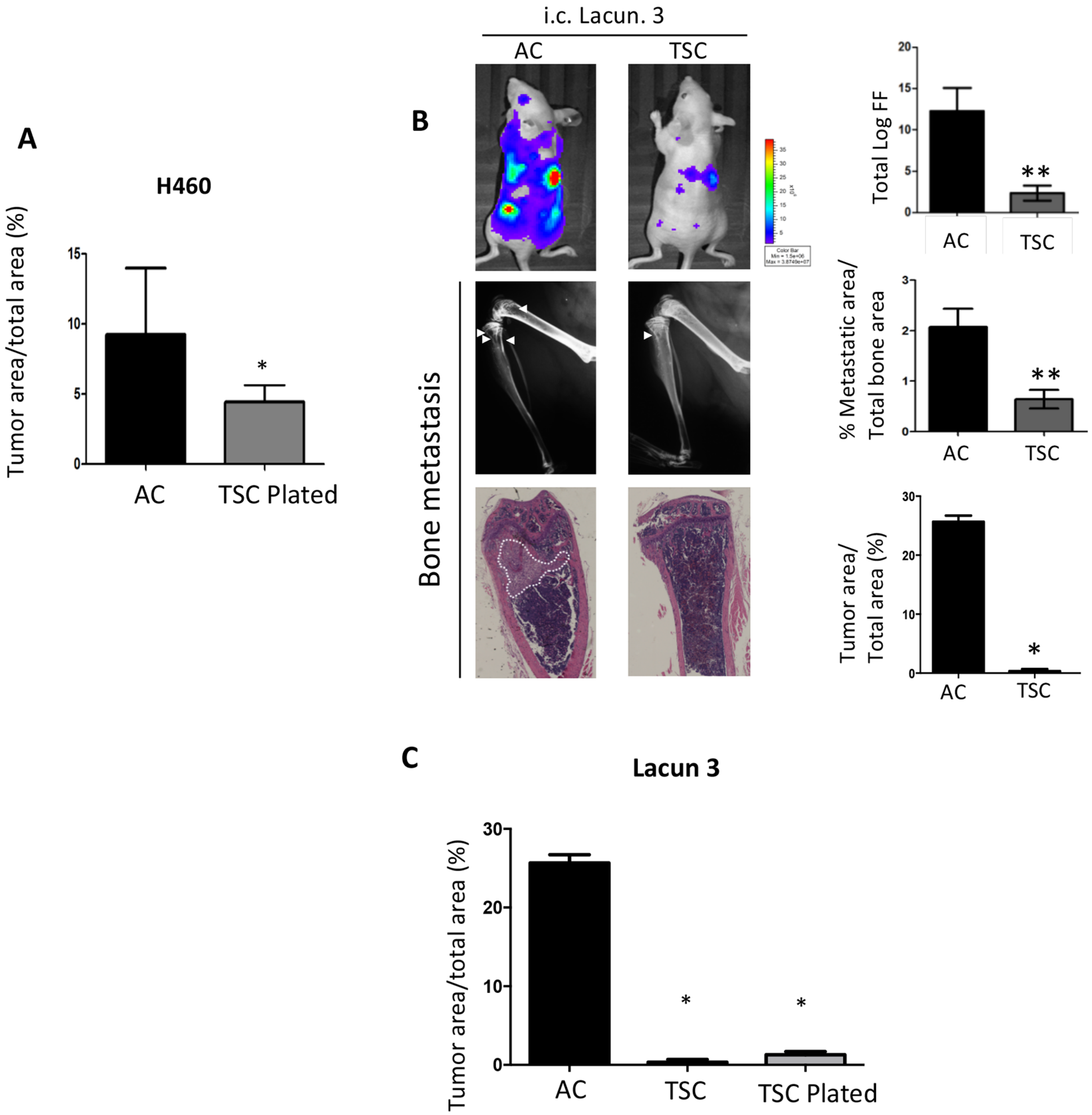

Supplementary Figure S4: Tumor sphere cultured (TSC) cells present lower metastatic ability than adherent cultured (AC) cells. A. I.c. inoculation of H460 cells produced lower percentage of bone tumor area/total area for mice injected with disaggregated TSC cultured for 24 hours (TSC plated) as compared to mice injected with AC cells. (*, $p < 0.05$), Error bars are mean ± SEM. **B.** Intracardiac inoculation (8 mice per group) of Lacun.3 cells produced lower percentage of bone tumor area/total area for mice inoculated with TSC and disaggregated TSC cultured for 24 hours (TSC plated) as adherent cells before their i.c. inoculation to AC cells. (*, $p < 0.05$), Error bars are mean ± SEM. **C.** Tumor burden in hindlimbs of mice i.c. inoculated with Lacun.3 cells.

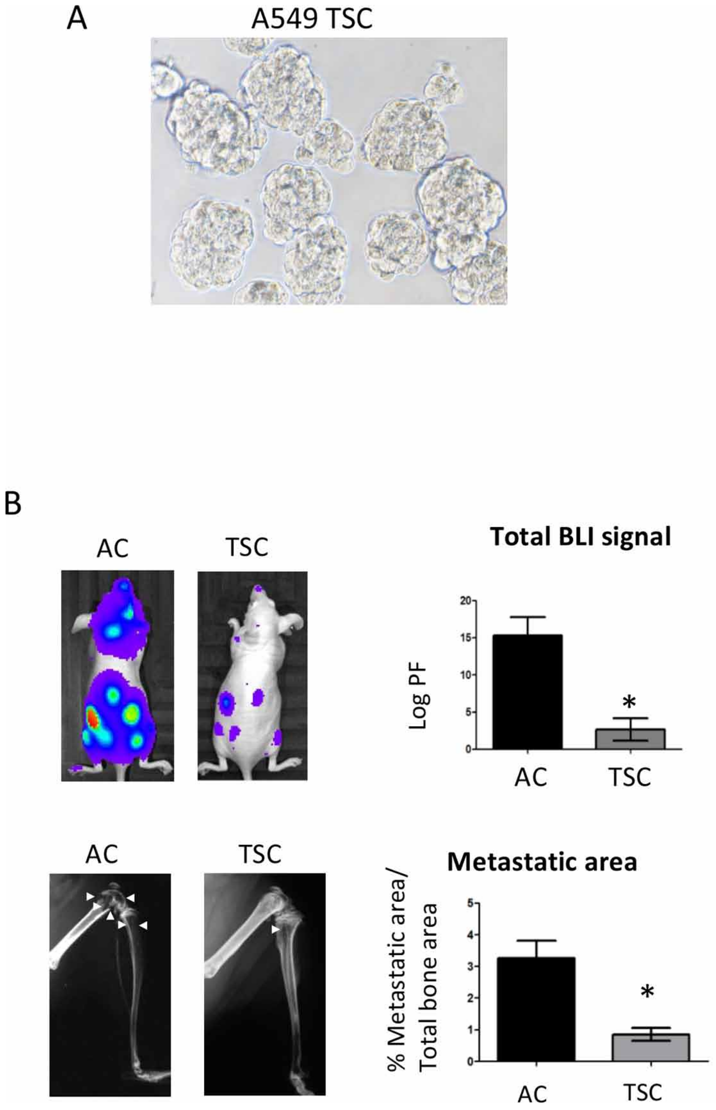

Supplementary Figure S5: Metastatic potential of A549 cells. A. Spheres prepared from the A549 cell line **B.** Intracardiac inoculation (8 mice per group) of tumor sphere cultured cells (TSC) led to lower BLI intensity and diminished osteolytic lesions adherent cultured cells (AC). (*, $p < 0.05$). Error bars are mean ± SEM.

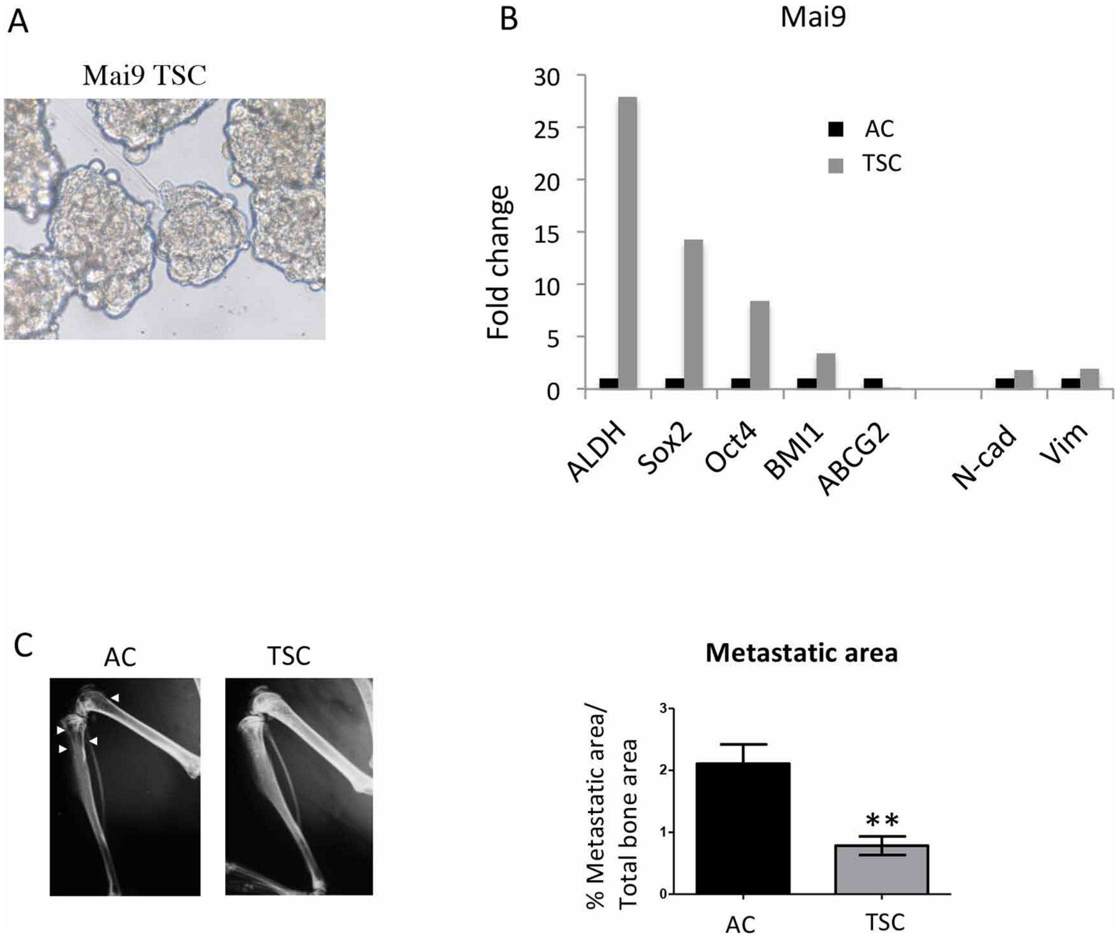

Supplementary Figure S6: Tumor spheres prepared from human malignant pleural effusion display lower metastasis ability. A. Pleural effusion from a patient (Mai9) form spheres when cultured in stem cell medium. **B.** These spheres present high mRNA levels of ALDH, Sox2, Oct4 and BMI1. **C.** Intracardiac inoculation (8 mice per group) of tumor sphere cultured (TSC) cells induced a reduced amount of osteolytic lesions as compared to adherent cultured (AC) cells. (**, $p < 0.01$), Error bars are mean ± SEM.

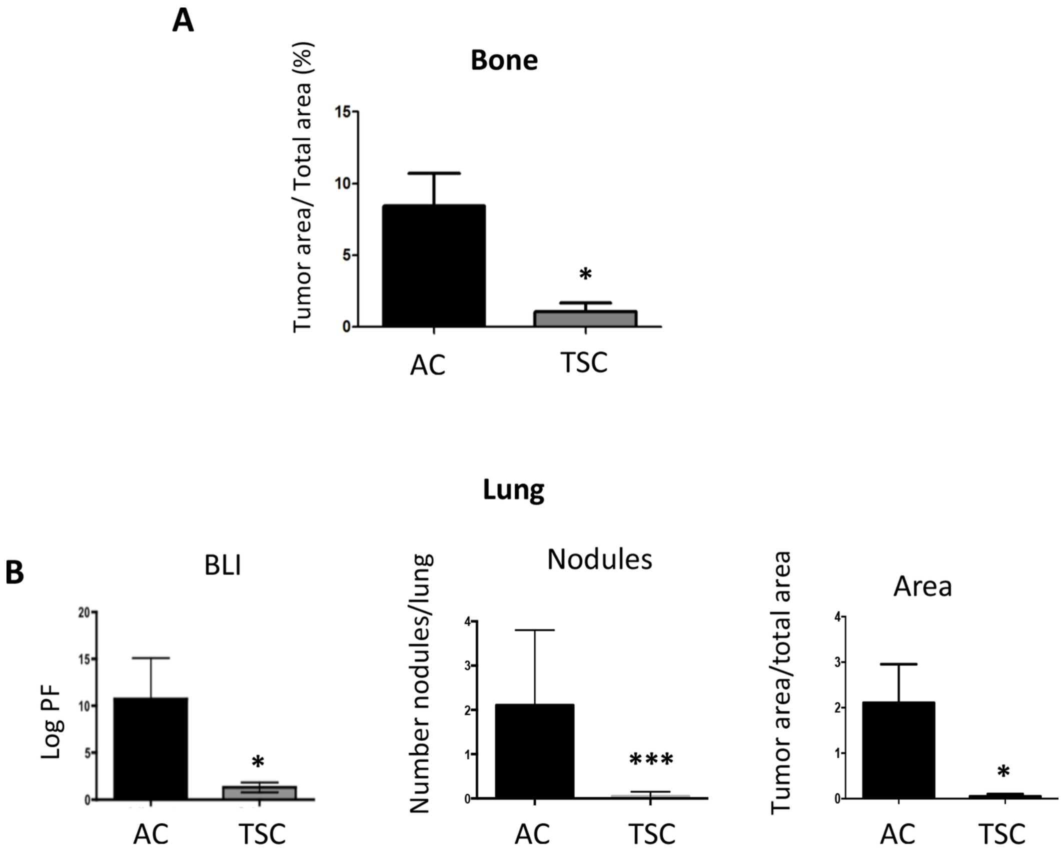

Supplementary Figure S7: Tumor sphere cultured (TSC) Lacun-3 cells display reduced bone colonization ability. A. Intratibial injection of tumor sphere cultured (TSC) Lacun.3 cells demonstrated reduced bone tumor area. **B.** Quantification of lung BLI as well as tumor area and the number of nodules per lung in mice injected i.t. with AC and TSC. (*$p < 0.05$, ***, $p < 0.001$), Error bars are mean ± SEM.

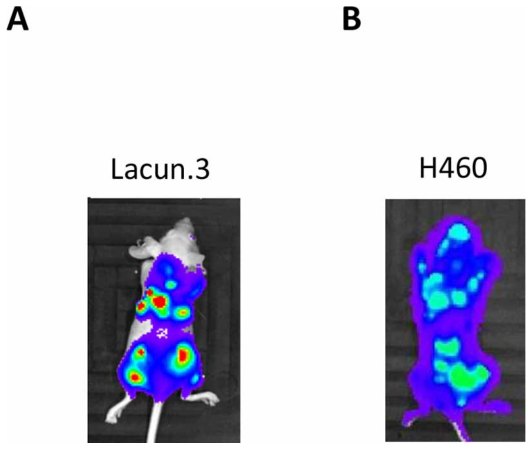

Supplementary Figure S8: Tumor metastases after i.c. inoculation of TSC derived cells. Representative BLI image of a mouse after i.c. inoculation of: **A.** Lacun.3 TSC cells at day 34 post-injection, and **B.** H460 TSC cells at day 21 post-injection, indicating that robust metastases develop over time.

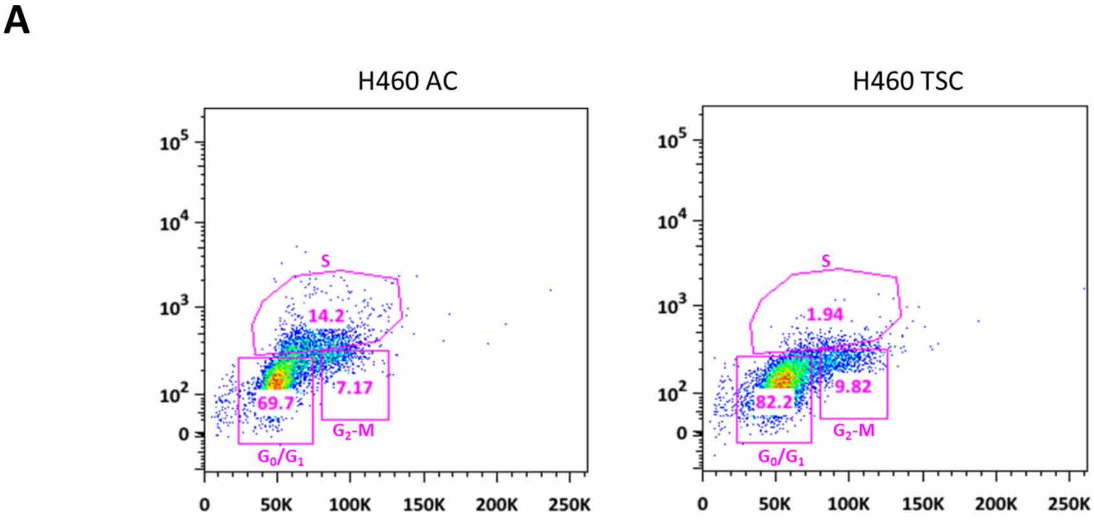

Supplementary Figure S9: BrdU assay. A. Percentage of positive BrdU cells was decreased when cells were cultured in sphere conditions (TSC) as compared to adherent (AC).

Supplementary Table S1:

Gene	Forward sequence	Reverse sequence
ALDH1	TCCTGGTTATGGGCCTACAG	CTGGCCCTGGTGGTAGAATA
ABCG2	CACCTTATTGGCCTCAGGAA	CCTGCTTGGAAGGCTCTATG
BMI1	ATGCAGCTCATCCTTCTGCT	GCATCACAGTCATTGCTGCT
CD44	AAGGTGGAGCAAACACAACC	AGCTTTTTCTTCTGCCCACA
ESA	TGTTGTGGTTCCGACTATAAACAG	GGGCTGCAACGTCATAATCT
MDR1	GCTCCTGACTATGCCAAAGC	TCTTCACCTCCAGGCTCAGT
OCT4	ACATCAAAGCTCTGCAGAAAGAACT	CTGAATACCTTCCCAAATAGAACCC
Sox2	AACCCCAAGATGCACAACTC	CGGGGCCGGTATTTATAATC
GAPDH	ACTTTGTCAAGCTCATTTCC	CACAGGGTACTTTATTGATG

Supplementary Table S2:

Gene	Forward sequence	Reverse sequence
ALDH1	GCACTCAATGGTGGGAAAGT	GGCCACACACTCCAATAGGT
ABCG2	TCGCAGAAGGAGATGTGTTG	TTGGATCTTTCCTTGCTGCT
b-catenin	GCAGCAGCAGTTTGTGGA	TGTGGAGAGCTCCAGTAC
Integrin a5b1	CACCATTCAATTTGACAGCAA	TCCTCTCCCTTGGCACTGTA
OCT4	AGAGGGAACCTCCTCTGAGC	AGATGGTGGTCTGGCTGAAC
ScaI	CCATCAATTACCTGCCCCTA	TTCCTGGCAACAGGAAGTCT
Tenascin C	ACTGTCTTGTGTGGTTTCAG	ATCCACAGTCACCATGGAC
Vimentin	TTCTCTGCCTCTTCCAAACTTTTC	GGGTATCAACCAGAGGGAGTGA
GAPDH	ACTTTGTCAAGCTCATTTCC	TGCAGCGAACTTTATTGATG
